# Assembly and mechanisms of bacterial type IV secretion machines

**DOI:** 10.1098/rstb.2011.0207

**Published:** 2012-04-19

**Authors:** Ellen L. Zechner, Silvia Lang, Joel F. Schildbach

**Affiliations:** 1 Institute of Molecular Biosciences, University of Graz, Humboldtstrasse 50/I, Graz 8010, Austria; 2 Department of Biology, Johns Hopkins University, Mudd Hall 235, 3400 North Charles Street, Baltimore, MD 21218, USA

**Keywords:** bacterial type IV secretion, horizontal gene transfer, bacterial conjugation, effector protein, pilus, core complex

## Abstract

Type IV secretion occurs across a wide range of prokaryotic cell envelopes: Gram-negative, Gram-positive, cell wall-less bacteria and some archaea. This diversity is reflected in the heterogeneity of components that constitute the secretion machines. Macromolecules are secreted in an ATP-dependent process using an envelope-spanning multi-protein channel. Similar to the type III systems, this apparatus extends beyond the cell surface as a pilus structure important for direct contact and penetration of the recipient cell surface. Type IV systems are remarkably versatile in that they mobilize a broad range of substrates, including single proteins, protein complexes, DNA and nucleoprotein complexes, across the cell envelope. These machines have broad clinical significance not only for delivering bacterial toxins or effector proteins directly into targeted host cells, but also for direct involvement in phenomena such as biofilm formation and the rapid horizontal spread of antibiotic resistance genes among the microbial community.

## 1. Introduction

The type IV secretion systems (T4SSs) are divided into three groups according to their function. The first group transfers DNA from one cell to another in a process called conjugation. Conjugation greatly increases prokaryotic genome plasticity, and has enormous importance in human health care as a major vehicle of antibiotic resistance spread among pathogens and commensal bacteria alike. Mobilization en bloc of large gene regions can efficiently transfer entire arsenals of functional modules that promote survival and infection activities. The T4SS of *Agrobacterium tumefaciens* resembles conjugation systems and functions to genetically transform plants. Delivery of nucleoprotein complexes to recipient cells leads to integration of the bacterial DNA into the host chromosome. Expression of these oncogenic bacterial genes induces tumour formation in a broad range of plant hosts [1].

A second pilus-based subgroup of T4SSs transfers protein. This process also depends on direct cell contacts between donor and recipient cells. A variety of bacteria that intimately interact with eukaryotic hosts—both pathogens and symbionts—have adopted T4SSs to transmit bacterial proteins to the host cytoplasm. The best-studied examples are used by Gram-negative pathogens. Some establish interaction between the pathogen and the host. Others inject one or several effector proteins into host cells, where they subvert multiple cellular functions to benefit the infecting pathogen [2].

Contact-independent secretion of protein is also part of the T4SS repertoire as exemplified by the secretion of pertussis toxin by *Bordetella pertussis* [3]. The T4SS of *Neisseria gonorrhoeae* secretes DNA into the extracellular environment instead of a recipient cell [4] using machinery evolutionarily related to conjugation systems. The mobilized DNA is not naked but appears to be linked to a leader protein with homology to conjugative relaxases [5].

Other related transfer systems, such as ComB of *Helicobacter pylori*, mediate DNA uptake from the environment leading to bacterial transformation [6]. Both dissemination and transformation processes play crucial roles in the genetic interaction network of the prokaryotic community.

Most T4SSs comprise three functional substructures: cell surface pili or adhesins that mediate contact between cells, a transport channel that conducts substrates across the bacterial cell envelope, and a type IV coupling protein (T4CP) that acts as substrate receptor at the cytoplasmic entrance of the secretion channel. T4CPs mediate multiple protein–protein interactions with cytoplasmic and inner membrane (IM) components of the secretion system. ATPase activity is associated with the release and unfolding of complexes between substrates and specific chaperones and is required to energize the secretion process.

The purpose of this review is to update information on the mechanisms employed by these systems. We focus on Gram-negative paradigms, including recent advances on structures of the protein assemblies involved. Crystal structures are available for the T4CP, several secreted proteins and essential T4SS components, as are high-resolution images of the supramolecular assembly of the envelope-spanning secretion channel. The electron microscopic structure of the T4SS core complex and the atomic resolution structure of its outer membrane (OM) pore have profoundly altered our understanding of these remarkable machines. Thus far, progress in understanding the mechanistic principles of T4SSs is due to extensive research of conjugation systems and the related DNA delivery system of *A. tumefaciens*. This archetypal system is composed of 12 components, VirB1–VirB11 and VirD4, which we will refer to throughout the review as VirB/VirD4. Most Gram-negative T4SSs are composed of phylogenetically or functionally related proteins.

## 2. Overview

### (a) Type IV secretion system subfamilies, functions and mechanistic principles

#### (i) Conjugation systems

**Figure 1.**
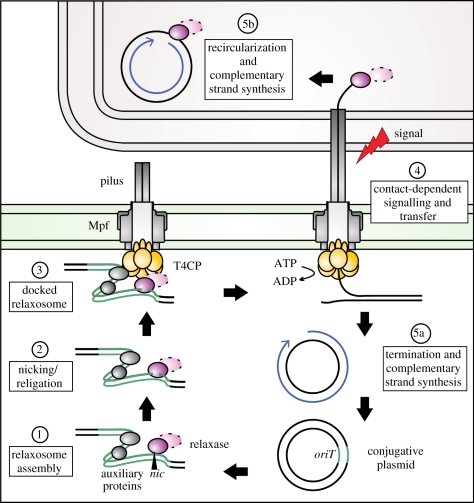
General mechanism of plasmid transfer. Conjugative plasmids carry an *oriT* and express proteins for pili, the envelope spanning T4 channel (Mpf), a T4CP (yellow), the secretion initiation complex (relaxosome), and secretion substrate and DNA nicking relaxase (pink). Some bifunctional relaxases are fused to a helicase or primase domain to facilitate conjugative DNA processing (dotted oval). T-DNA is processed as described in the text, and transferred as ssDNA. The complementary plasmid strand remains circular throughout. For simplicity, stages 1–4 of plasmid DNA processing are illustrated with linear DNA fragments.

Conjugation systems are the largest and most widely distributed of the T4SS subtypes. These systems are responsible for plasmid conjugation in Gram-negative and Gram-positive bacteria, as well as the transfer of integrated conjugative elements, which are phage-like sequences that have been integrated into the bacterial chromosome [7]. Plasmids of Gram-negative bacteria are the best-studied paradigms. These large, extrachromosomal genetic elements are self-transmissible: plasmids encode the entire machinery they need for transfer to another host. Multi-protein assemblies involved include the mating pore formation (Mpf) proteins, which provide a cell envelope-spanning transport channel. The core structure of this apparatus is shared throughout the T4SS family. Surface filaments known as pili establish contact between cells. The general mechanism of plasmid transfer is well-characterized (figure 1). (i) Multiple proteins assemble on the plasmid origin of transfer (*oriT*) to form the relaxosome. (ii) This stable complex prepares the single-strand of plasmid DNA destined for transfer (T-strand) via the nicking activity of a relaxase enzyme. Initiation of transfer requires cleavage of the phosphodiester bond at a specific position, *nic*, within *oriT.* The reaction is mediated by a tyrosine residue of the relaxase, so that a covalent tyrosinyl-DNA adduct is formed. (iii) This nucleoprotein conjugate is specifically recognized by the plasmid-encoded T4CP. (iv) In response to contact-dependent initiation signals, relaxase linked transferred DNA (T-DNA) is probably actively pumped through the transport apparatus. (v) In the recipient following termination of transfer, the nicking reaction reverses, yielding the original circular plasmid molecule and freeing the relaxase. (vi) Finally, stabilization of the original plasmid DNA strands by conjugative replication occurs in both donor and recipient cells. Direct cell contact is required for conjugative transfer. Whether or not the genetic material is conveyed through the pilus lumen remains unresolved (see §3).

The extensively studied VirB/VirD4 system of *A. tumefaciens* is related to conjugation paradigms [8]. The range of virulence (Vir) proteins and the T4SS required for DNA delivery and tumour formation are encoded on a tumour-inducing (Ti) plasmid of virulent *Agrobacterium* strains. The Ti plasmid is also a source for the T-DNA delivered with additional Vir proteins to the plant cell cytosol. The Vir proteins suppress the host innate immune system and support the transfer, nuclear targeting and integration of T-DNA into the plant chromosome. Expression of T-DNA genes results in the synthesis of plant hormones, which cause tumour growth, and the biosynthesis of amino acid–sugar conjugates called opines, which provide a specific food source for the pathogen. Research with this model system has been profoundly important, not only for understanding host–pathogen recognition and targeted macromolecular delivery, but also in the development of plant genetic engineering technologies.

**Figure 2.**
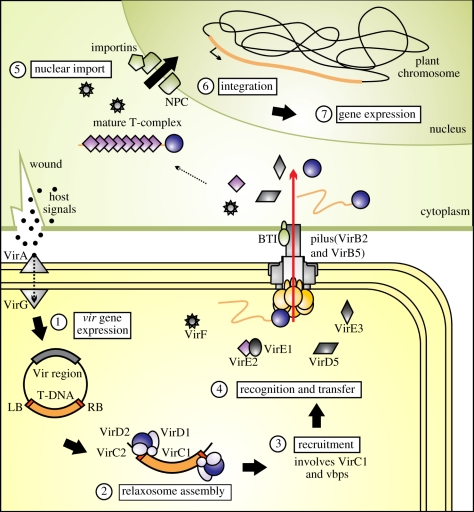
Stages of *Agrobacterium tumefaciens* T-DNA delivery to plants. (1) The VirA/VirG system induces Ti plasmid *vir* gene expression in response to signal molecules at wound sites. (2) VirD2 relaxase and relaxosome components excise T-DNA at *oriT*-like border sequences. (3) Recruitment of the relaxase T-DNA intermediate to the T4SS involves binding proteins (vbp) and spatial determinant VirC1. (4) T4CP VirD4 (yellow) recognizes protein and nucleoprotein secretion substrates and initiates transfer. Pilin VirB2 contacts plant receptor proteins (BTI). Delivery of bacterial effectors to plant cytosol is followed by VirE2 T-complex maturation, (5) nuclear import, (6) integration and (7) transgene expression.

**Figure 3.**
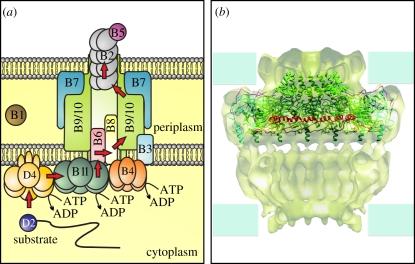
General translocation pathway and core structure of a T4SS. (*a*) The translocation pathway of substrates through the *Agrobacterium* VirB/D4 system. For a detailed description see text. (*b*) A cutout of the Type IV secretion system core complex and crystal structure of the O-layer. The cryo-EM structure (surface representation) of the T4S system core complex is here superimposed with the crystal structure of the O-layer. For the crystal structure, VirB7, the C-terminal domain of VirB9 and the C-terminal domain of VirB10 are shown in magenta, cyan and green. The lever arm sequence of VirB10 is shown in red. Figure contributed by Drs Karen Wallden and Gabriel Waksman.

The *A. tumefaciens* VirB/VirD4 system serves as a convenient prototype for type IVA (F-like and P-like) conjugation systems, as these typically have closely related homologues of most or all of the components. The *A. tumefaciens virB* operon encodes VirB1 through VirB11. Analogous to conjugative Mpf proteins, these are required for secretion channel assembly. The principal mechanisms involved in T-DNA delivery to plant cells have been established (figure 2). Bacterial cells contact a host-cell receptor using VirB2 pilin and VirB5 adhesin. In the donor, the T4CP VirD4 delivers relaxase-linked single-stranded T-DNA to the IM secretion channel. Other bacterial factors specifically transported include VirE3, VirF, VirD5 and the single-stranded DNA (ssDNA)-binding protein VirE2, which coats and protects the T-DNA. VirD4-like proteins have DNA-dependent ATPase activity, and current models propose that DNA translocase activity ensures processivity during secretion. Two additional ATPases energize the system from the cytoplasm. VirB4 is present in all known Gram-negative T4SSs, whereas VirB11 is a hexameric ATPase not universally found. These ATPases are thought to provide energy for T4SS assembly and active secretion. Seminal work by Cascales & Christie [9] developed a formaldehyde cross-linking assay to identify the sequential translocation steps occurring during substrate secretion, providing the first clearly defined pathway for substrate progression through a T4 secretion apparatus (figure 3*a*). The VirD4 T4CP receptor binds the substrate first and transfers it to the VirB11 ATPase. The ATP hydrolysing activity of the VirD4, VirB4 and VirB11 trio is coordinated by VirB11 to transfer the substrate to channel proteins VirB6 and VirB8, conveying the substrate across the IM. The substrate then contacts VirB9 and VirB2 within the channel for subsequent translocation across the periplasm and OM. This pathway is likely to be conserved among DNA delivery systems. The question of whether other T4 functional subgroups operate similarly awaits analysis of additional systems at this level of experimental detail.

#### (ii) Effector protein translocation systems

Another large subgroup of the pilus-based T4SSs is dedicated to translocating effector proteins directly to the cytosol of eukaryotic cells. The systems are ancestrally related to conjugation systems, yet have adopted highly diverse functions. These systems are important to the infection strategies of many bacteria, including human pathogens *H. pylori*, *Legionella pneumophila*, *Bartonella* spp. and *Brucella* spp. Secretion enhances bacterial colonization and promotes survival within the cells or tissues of an infected host.

The variety of hosts and the infection strategies pursued by these pathogens are highly diverse, with a corresponding heterogeneity in the primary and higher order structures of system components and in the macromolecules secreted. In addition to the conventional type IVA T4SSs, a distinct type IVB secretion family comprises systems resembling I-like conjugation machinery. Type IVB T4SSs are found in intracellular bacterial pathogens such as *L. pneumophila*, *Rickettsiella grylli* and *Coxiella burnetii*. A genetic locus variously designated as *d*efect in *o*rganelle *t*rafficking (*dot*) or *i*ntra*c*ellular *m*ultiplication (*icm*) encodes a T4SS involved in establishing a replicative niche, intracellular replication or macrophage killing [10]. The Dot/Icm system exhibits remarkable versatility in its capacity to translocate over 140 distinct effector proteins to eukaryotic host cells, and to mediate conjugative mobilization of IncQ plasmid DNA. A growing volume of bacterial genomic information implies that the type IVB family is not limited to *Legionella* and related bacteria [11]. Diversity in system components and functions is illustrated also by *H. pylori.* This pathogen uses the *c*ytotoxin-*a*ssociated *g*ene (Cag) T4SS for host-cell interactions, and other T4SSs for horizontal gene transfer. The Cag–T4SS injects a single effector protein, inducing a pro-inflammatory response and multiple cytoskeletal and gene regulatory effects in gastric epithelial cells [12]. Of the more than 14 components of the secretion apparatus, only a few *cag* genes encode proteins with clear sequence similarities to T4SS prototypes, suggesting potential differences in channel architecture, details of assembly and the mechanisms of secretion.

Despite the attendant energetic costs, many bacteria maintain more than one T4SS, suggesting these systems contribute significantly to the evolutionary success of the organism. Frequently, distinct T4SSs are used for horizontal gene transfer and for interactions with various host cells. Moreover, lateral acquisition and establishment of multiple virulence systems occur, enabling the T4SSs to adopt highly diverse functions during infection. As an interesting example, the accessory genome of *Bartonella* encodes at least three distinct T4SSs, each contributing to infection and survival processes in unique ways [13,14]. This genus comprises arthropod-borne pathogens that colonize endothelial cells and erythrocytes of their mammalian reservoir hosts [15]. *Bartonella* species of the modern lineage encode one or both of the closely related VirB-like T4SS, VirB/VirD4 and Vbh [16]. Several effector proteins translocated by these independent systems are key to establishing chronic infection [13]. The third T4SS, Trw, does not transmit bacterial effectors but produces several pilus-associated surface proteins critical to the invasion of erythrocytes [17,18]. The *Bartonella* strategy uses T4SS for pathogenesis. Yet, the diversity appears to promote long-term fitness, by conferring host adaptability, which results in reduced virulence properties in a given host, and in the adaptation to novel hosts.

#### (iii) Contact independent type IV secretion system

The third subgroup exchanges macromolecules between cell and external milieu. This includes the *p*ertussis *t*oxin *l*iberation system (Ptl), which secretes a complex protein toxin across the cell envelope of *B. pertussis* to the cell exterior [19,20]. Secretion of the toxin proceeds via a two-step mechanism. Only nine *virB* homologues are present in the Ptl machinery. VirB1, VirB5 and the T4CP VirD4 are absent. Without a dedicated T4CP, translocation of the pre-proteins as individual subunits across the IM apparently relies on the general Sec secretion pathway [21]. Once they reach the periplasm, the subunits associate with the OM and disulphide bond formation stabilizes the holotoxin. For the assembled holotoxin to traverse the OM, it must enter the Ptl secretion channel from the periplasm. The route of entry is not known, nor is it understood how this process is controlled. Resident periplasmic proteins, which can be much smaller than the holotoxin, fail to escape the cell.

DNA export by the T4SS of *N. gonorrhoeae* relies on a specific *oriT*-like sequence, a relaxase-like protein and a T4CP [22]. Unlike conjugation, transfer initiation occurs in the absence of cell contact. Like conjugation, however, the process involves recognition of dsDNA and translocation of the ssDNA form. Natural transformation of *H. pylori* is exceptional because it involves a T4SS ComB [23,24]. Recent evidence indicates that DNA uptake occurs in two steps: the ComB apparatus conveys external dsDNA across the OM to the periplasm. From there, transfer to the cytoplasm apparently occurs via the ComEC channel conserved in all known transformable bacteria [25]. A single ATPase, ComB4, may provide energy for system assembly or for substrate translocation across the OM. DNA is thought to enter the cytoplasm in single-stranded form but both the mechanism of conversion and the motor protein of the IM step remain unknown.

## 3. Functional assemblies and their architecture

### (a) The core complex

T4SSs each possess an envelope-spanning channel composed of conserved components termed the core complex. Early biochemical studies showed that VirB7, VirB9 and VirB10 form a transporter subassembly that is both intrinsically stable and stabilizing for other VirB subunits. This core complex from the conjugative pKM101 system provided the first high-resolution images [26]. The core secretion channel is a multimeric VirB7–VirB9–VirB10 complex containing 14 copies of each protein. A cylindrical structure spanning the entire cell envelope is composed of two layers (designated I and O). Each layer forms a double-walled ring-like structure that defines hollow chambers inside the complex (figure 3*b*). The structure surrounds a central chamber of about 80 Å at its widest point. The N-terminal domains (NTDs) of VirB9 and VirB10 comprise the I layer and this part of the channel is anchored in the IM by an N-terminal transmembrane helix of VirB10. An opening at the base of the I layer spans 55 Å. The O layer consists of a main element and a narrower cap that is constricted to a 10 Å opening, too small to let substrates through. VirB8 interacts with several VirB proteins providing a key nucleation effect important to both assembly and stabilization [27–30]. A plausible model predicts that a complex of VirB8, VirB6 and the colocalized ATPases VirB4, VirB11 and VirD4 create an IM translocase within the opening formed by the VirB10 scaffold, but this requires further study.

A crystal structure of the OM-spanning part of the core complex, including the entire O layer, was determined at 2.6 Å resolution [31]. The cap structure, composed of a hydrophobic ring of C-terminal two-helix bundles contributed by VirB10, forms a 32 Å channel across the OM that protrudes to the cell surface. The cryo-electron microscopic and the crystal structures differed in the inner diameter dimensions of the pore. The observed variation—from 32 Å to a constricted form 10 Å across—implies that VirB10 controls OM pore opening. The crystal structure of the OM complex reveals that VirB10 is endowed with a long peptide sequence located between its N-terminal domain (that is part of the I-layer) and its C-terminal domain (that forms the O-layer and the pore). This sequence forms an extended arm (which was termed ‘lever arm’) that makes extended contacts with three adjacent subunits within the tetradecameric assembly. All together, these 14 lever arms form a shelf around the inner surface of the channel and it was observed that this shelf is at two different positions in the electron microscope structure of the core complex (down) compared with the OM complex X-ray crystal structure (up). It was then suggested that VirB10 exerts control of pore opening/closing through the positioning of the lever arm shelf. VirB10 undergoes a structural transition when it senses modulation of the ATP-bound state of VirB11 or VirD4 [32]. Accordingly, VirB10 is proposed to transduce energy from these ATPases into conformational changes in the channel during active secretion. Because VirB10 also forms the OM pore, one can easily envisage a mechanism whereby conformational changes in ATPases are directly transmitted to VirB10, resulting in VirB10 pore opening or closure with the VirB10 lever arms acting as relays. Recent evidence supports the model that the C-terminus of VirB10 is important to regulating passage of substrates across the OM [33].

Given the broad phylogenetic conservation of core components, it is reasonable to propose that T4SSs share a common central structure for secretion while peripheral components involved in specific interactions with substrates and target cells have diverged accordingly. As an example, the conjugative Trw system of R388 and Trw of *Bartonella* spp., involved in erythrocyte attachment, share high sequence similarity and genetic organization. Functional exchangeability was confirmed for the core proteins but not for components of the pilus [34]. Similarly, the minor T-pilus component VirB5 can be partially complemented for pilin export by pKM101 homologue TraC, but the complemented strain remains avirulent [35]. Genetic experiments with *A. tumefaciens* VirB/VirD4 support the model that two types of core structures exist based on the associated peripheral proteins: one suitable for protein secretion, and the other dedicated to T-DNA transfer [29,32,36,37].

Further variation in the T4SS channel architecture, peripheral functions and details of the biogenesis process is anticipated as research with known systems progresses and as novel, unrelated systems are identified. Heterogeneity is discernible in the Cag–T4SS of *H. pylori* [38]. Clear similarity exists for Cag proteins to VirB9, VirB10, VirB4, VirB11 and VirD4. Topology predictions, biochemical and functional studies support the assignment of further VirB homologues, and finally several additional Cag proteins are unique to this secretion system. Deviation from the pKM101 suprastructure is anticipated [39]. Localization and interaction studies of Cag proteins revealed the basic core architecture is elaborated with additional components apparently important to oligomerizing and stabilizing the OM-associated subcomplex [40,41].

The F plasmid encodes about 40 genes dedicated to secretion; 18 of these are involved in assembling an active system. Eight Tra proteins correspond to widely conserved members of T4SSs (TraA, -B, -C, -E, the N-terminal portion of TraG, -K, -L and -V) [42]. Nine additional auxiliary factors are specific to F-like T4SSs. These and two other factors unique to F (TraQ and TraX) contribute to the processes of pilus assembly, pilus dynamics and mating pair stabilization that are hallmark features of the F system, but dispensable to others of the T4 group [42]. Six of the auxiliary factors required to assemble F-pili form a cluster of interacting proteins in the periplasm [43]. TraH is the most highly connected node of this group as defined by two-hybrid analysis. At present, little is known about the architecture of their peripheral positions relative to the TraB–TraV–TraK core structure [44], but evidence for physical association of at least one of these factors with the core structure has been obtained [45].

### (b) Extracellular pili

Conjugative pili have been studied for over 40 years [45,46]. Accordingly, these are the best understood of the T4SS surface organelles. Pili are usually long thin filaments extending from the surface of donor cells as tube-like structures with an outer diameter of 8–10 nm and a central hydrophilic lumen of 2–3 nm [47–50]. In the systems examined, pili contain one quantitatively predominant subunit. The first step in pilus production is post-translational processing of propilins. Removal of the F propilin leader peptide is followed by acetylation. The P-like pilins of RP4 (TrbC) or Ti (VirB2) undergo N- and C-terminal truncation. Subsequent cyclization creates unique intramolecular cyclic peptides of exceptional resilience and stability [51,52]. Pili are assembled by addition of pilin subunits at the cell-proximal end [53,54]. The numerous factors essential for F pilus biogenesis can be distinguished on two functional levels: those that promote pilus tip formation and those that allow pilus extension [55]. Stages of T-pilus assembly are becoming apparent [28,29]. Pilus biogenesis requires energy, probably provided by the VirB4-like proteins [53,56].

Recent studies by Clarke *et al*. [53] applied live cell imaging to directly visualize the extension and retraction cycles of F-pili. Elongating F-pili can switch to retraction but the opposite dynamic has not been observed [45]. Filaments retract processively from the surface of the cell and pilin subunits return to the membrane. Remarkably, F-pili appear to rotate about their long axis during growth. Constraint of the cell-distal end by surface or cellular contacts can actually lead to the development of supercoils owing to torsional stress. The properties of pilus flexibility and dynamic movement conferred by the F-specific extra contingent of genes may be uniquely required for cell-to-cell transfer in fluid environments. Silverman & Clarke [45] propose that F-pili function as sensory organelles that sweep out a large volume around the donor cell in search of suitable recipients for gene transfer. In comparison, consistent with the ecological niche, bacteria relying on most other conjugative systems express fewer components, elaborate shorter rigid pili and establish productive intercellular contact only on solid surfaces [57].

### (c) Type IV coupling protein

A third class of conserved components, the T4CPs, is not required for system biogenesis, but is essential for active secretion. T4CPs are associated with nearly all T4SSs. The typical T4CP has an N-terminal transmembrane domain, and a large cytoplasmic domain containing a membrane-proximal nucleotide-binding domain. Conserved nucleotide-binding motifs are essential to secretion in most systems. Mutations in these motifs typically evoke a dominant negative phenotype, implying that T4CPs function as multimers, consistent with biochemical studies of Gram-negative prototypes. A soluble fragment of TrwB from plasmid R388 was crystallized and shown to adopt a ring-shaped structure similar to RecA and DNA ring helicases with a diameter of 110 Å and height of 90 Å [58]. The channel diameter in the centre of the hexamer ranges from 20 Å to an approximately 8 Å constriction at the cytoplasmic pore. T4CPs are anchored to the T4SS core structure via interactions with the NTD of VirB10 [59,60]. Several T4CPs have demonstrated binding to DNA, relaxase components and secreted effector proteins, and thus are generally attributed the role of recognizing and delivering substrate to the secretion apparatus to initiate transfer. Current models based on evidence from DNA-mobilizing systems project that ATP hydrolysis by T4CPs energizes substrate movement [9,61].

## 4. Initiation pathway

### (a) Substrate processing

Relaxases belong to a broad family of nucleases with a conserved His–Hydrophobe–His motif [62]. Structural data confirm that the motif coordinates a metal, likely Mg^2+^, essential for DNA cleavage [63–66]. In most relaxases studied, a third His also ligands the metal, whereas a His–Glu–Asn trio serves the purpose in MbeA from plasmid ColE1 [67]. Judging from the F TraI relaxase:ssDNA complex, the bound metal coordinates oxygens of the scissile phosphate, serving to both position the phosphorus and render it relatively more electropositive for nucleophilic attack [65]. The neutral His, unlike acidic residues, does not substantially reduce the metal's charge, rendering it more effective in polarizing the phosphorus atom. Relaxases usually have multiple Tyr residues in the active site, often in flexible regions. This flexibility has been invoked in models of sequential cleavage by different TrwC active site Tyr residues [68], and to account for the fact that multiple Tyr are competent to cleave or ligate an *oriT* sequence [66,69].

In the relaxase-nicking reaction, an active site tyrosyl hydroxyl attacks the scissile phosphate, releasing the 3′ bridging oxygen and forming a long-lived ssDNA–protein conjugate. This intermediate protects the 5′ phosphate of the T-DNA as it enters the recipient, links the ssDNA to the translocation signal (TS) in the relaxase to target the ssDNA for transfer, and provides the means to rejoin the plasmid ends in the recipient. The covalent linkage, combined with the observed extensive contacts between relaxase and ssDNA [64,65,70], accounts for the remarkable stability of some relaxase–ssDNA complexes, a phenomenon noted early on by Kline & Helinski [71]. This trait is not universal: pCU1 TraI demonstrates moderate affinity and specificity [66]. DNA conformation can also play an important role in recognition by TrwC and F TraI, with base–base interactions near *nic* helping define specificity and position the scissile phosphate for cleavage, and hairpins more distal improving affinity [64,65,70,72,73].

### (b) Colocalization of substrates, type IV coupling proteins, type IV secretion system

Tracking of plasmids F and R751 *in vivo* demonstrated that these plasmids are localized to the centre or the one-quarter and three-quarter cell positions [74,75]. As pili are not restricted to these positions, the plasmid–relaxase conjugate must be recruited to the conjugative machinery to initiate transfer. Little is known about how the many T4 channel proteins, T4CP and relaxosome factors colocalize in the cell. Mechanistic detail is emerging from the *A. tumefaciens* system (figure 2). The Ti plasmid-encoded MinD-like ATPase VirC1 promotes relaxosome nucleation and processing of T-DNA borders [76]. Three host proteins (Vbp1, Vbp2 and Vbp3) are proposed to succeed VirC1 and coordinate colocalization of the T4SS cytosolic energizing components, the T4CP and the VirD2-processed T strands [77]. Moreover, the colocalization of VirB8 and the *A. tumefaciens* MinD helical scaffold imply that T4SS trafficking exploits the host's existing infrastructure for distribution [27].

### (c) Substrate presentation: signals and chaperones

In all T4SS subfamilies, the entry of macromolecules to the cell envelope-spanning secretion channel involves the T4CP. Rare exceptions are the virulence systems of *Brucella* spp. and *B. pertussis*. Identification of the appropriate macromolecules for secretion is selective and some knowledge of the underlying mechanisms is emerging. Proteins translocated by T4SSs are endowed with TSs that identify them as substrates for secretion. Vergunst *et al.* [78] applied the Cre recombinase reporter assay to map protein features that mediate their recognition. Data from the *A. tumefaciens* VirB/VirD4 paradigm and the *L. pneumophila* Dot/Icm T4SS revealed that a surprisingly simple cluster of positively charged or hydrophobic residues at the C-terminus constitutes a functional TS for secretion substrates [79,80]. The simplicity of recognition exhibited in these systems is probably not general, however. More complex bipartite signals are shared by the seven known VirB/VirD4-translocated *Bartonella e*ffector *p*roteins (BepA–BepG). The charged but not conserved C-terminal tails are combined with one or more copies of a second conserved *B*ep *i*ntracellular *d*elivery (BID) domain [81]. Modular architecture is a feature shared by the Cag–T4SS of *H. pylori*. The CagA effector carries a 20 amino acid C-terminal TS adjacent to a larger interaction domain for its secretion chaperone CagF [82]. Together, these features are sufficient to recruit CagA to the secretion apparatus. Relaxases carry comparatively large, complex signals. Mapping of the TS in F-like relaxases and MobA of R1162 revealed that each carries two distinct TSs at internal positions in the protein that are independently competent to mediate secretion by a conjugative T4CP [83,84]. The F-like TS module is conserved within relaxases of the MOB_Q_ and MOB_F_ groups.

T4SS can recognize and transfer proteins of highly related systems. This principle was exploited to identify effector proteins of the *Coxiella burnetii* system using the surrogate Dot/Icm system of *L. pneumoniae* [73]. Relaxases introduce an interesting variable among T4SS substrates in that secretion of the protein—covalently attached to DNA—conveys the genes for the secretion machinery itself into a variety of new hosts. The potential range of different systems resident in a single bacterium is quite variable as a result. Some conjugative and mobilizable relaxosomes show a narrow range of compatibility with components of heterologous systems for effective plasmid transfer [85,86]. In contrast, plasmids R1162 and RSF1010 exemplify factors that can be translocated by many different conjugative and effector T4SSs. MobA and other relaxosome proteins are thus remarkably promiscuous in successfully interacting with components of distantly related systems. Interestingly, secretion of MobA by the *A. tumefaciens* VirB system again relies on the generic cluster of positive charge at the C-terminus similar to other substrates of this system [80]. By contrast, the P-like transfer system specified by plasmid R751 required the larger MobA signals [83]. Although substrate recognition is highly specific, the T4CP can recruit its cognate substrate to a different T4SS via productive interactions with VirB10 [59]. Mobilizable plasmid CloDF13 encodes its own T4CP, which interacts with various transporters. VirD4 of *A. tumefaciens* effectively replaces conjugative T4CPs in plasmid transfer [87]. Remarkably, the R388 relaxosome is translocated by *Bartonella henselae* to both bacterial and human cells in a process resembling conjugation [88]. Transfer required the *virB* genes of the VirB/D4 T4SS and the conjugative relaxase-helicase TrwC. Coexpression of R388 T4CP TrwB facilitated transfer, but was not essential. Thus, recruitment of TrwC to the T4S apparatus was mediated by components of *Bartonella*. The C-terminal tail, and not the TS used by the cognate protein TrwB, may support transfer. In summary, relaxases of broadly disseminated plasmids appear to achieve wide recognition through modular displays of distinct recognition signals.

### (d) Substrate presentation: chaperones

**Figure 4.**
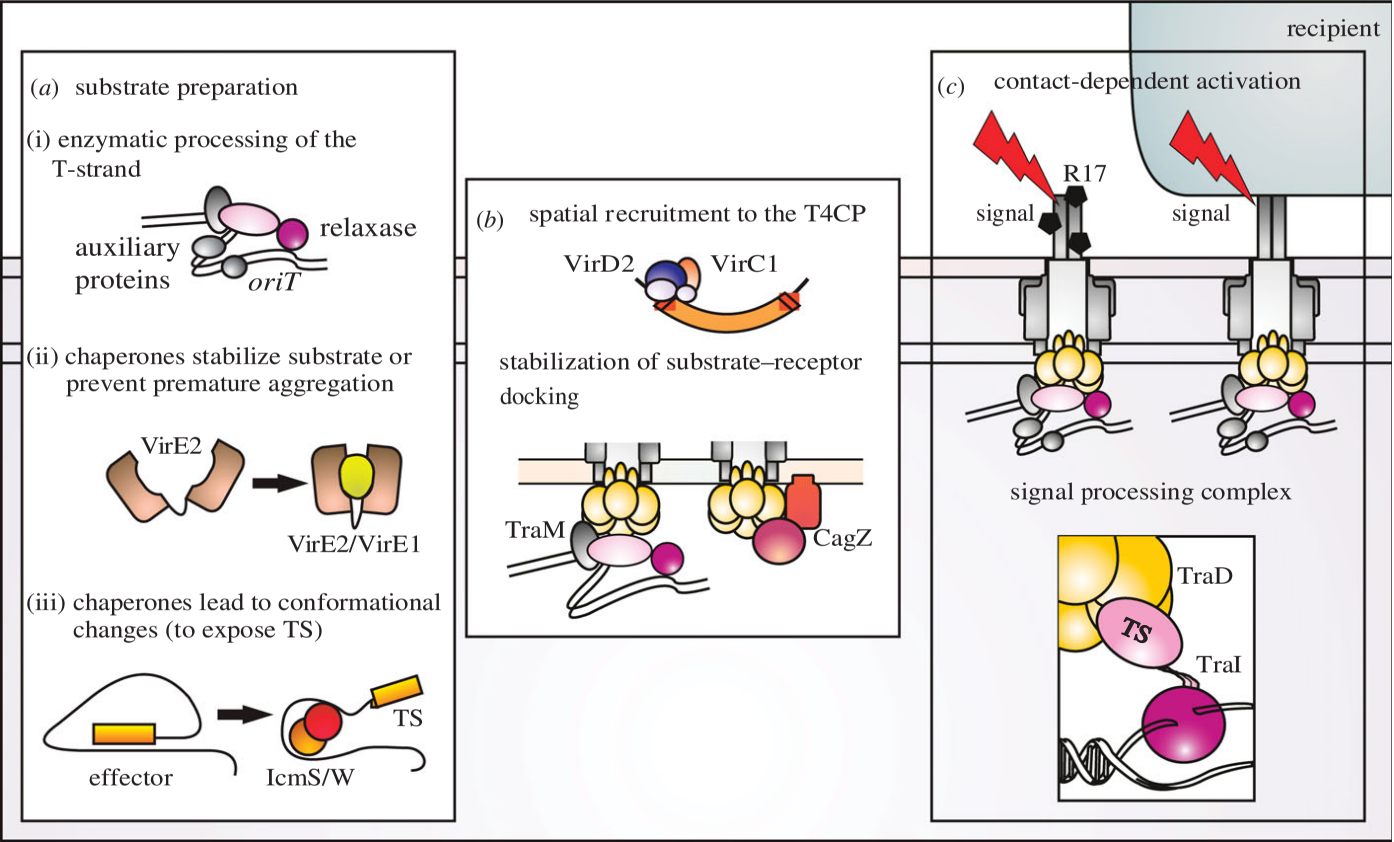
Functions of initiation factors in type IV secretion systems. (*a*) Functions in substrate preparation exemplified by (i) the conjugative relaxosome, (ii) VirE1_At_ chaperone–VirE2_At_ effector complex and (iii) IcmSW heterodimers complexed with *Legionella pneumoniae* effector protein. (*b*) Factors involved in spatial recruitment to the type IV coupling protein (T4CP; e.g. VirC1_At_), or stabilization of substrate–T4CP interactions (e.g. TraM_F_ or *Helicobacter pylori* CagZ). (*c*) Finally, exocellular signals induce transfer initiation presumably by controlling the conformation and ATPase activity of the T4CP. A receptor/signal processing complex for F-like T4SS activation is illustrated with the TraI_R1_ N-terminal domain linked to plasmid *oriT* and the T4CP via translocation signal.

Before entering the translocation channel, secretion substrates may form complexes with specific cytosolic binding partners that act as pilots, presenting effectors to the substrate receptor, and/or as masking factors to prevent premature aggregation of the substrate (figure 4*a*). Specific secretion chaperones have a central role in the secretion strategy of type III secretion systems. Examples of chaperone activities in T4SS are known, but this aspect has been understudied so far. Secretion substrates generally are thought to undergo at least partial unfolding to accommodate the narrow inner diameter of the envelope-spanning channel. It is anticipated that the unfolding process exposes region(s) that serve as secretion signal and as binding domain for a cognate chaperone. The interaction stabilizes intermediate stages of folding, protects from premature aggregation, and displays TS for specific recognition. In *A. tumefaciens*, VirE1 prevents VirE2 from forming oligomers and from aggregation. Three T4SS chaperones (IcmS, IcmW and LvgA) of the Dot/Icm effector translocation system have been characterized [89]. Heterodimers of IcmS with either partner protein form chaperone complexes with effector proteins. Induced conformational change in the bound effector unmasks the TS for recognition (figure 4*a*).

Specific intracellular interactions among components of T4SSs are often revealed through mutual stabilization—the loss of one partner results in destabilization of another. This approach has been used extensively to detect interaction partners for the T4CP and other components of the Dot/Icm system. T4CPs within the type IVB subgroup include DotL or IcmO of *L. pneumophilia* and TrbC in I-like conjugation systems. Cellular concentrations of DotL and DotM (I-like TrbA) are much reduced in *L. pneumophilia* mutants lacking DotL, DotM or the IM protein DotN [90]. Complex formation between these factors is thus predicted.

The Cag–T4SS of *H. pylori* includes three predicted cytoplasmic or IM components, which are essential for CagA translocation but not for inducing chemokine secretion from gastric cells and thus probably not for assembling the secretion apparatus. Cagβ (HP0524) is related to T4CPs [91–93]. The translocation accessory factors (CagF and CagZ) contribute chaperone-like roles. CagF was detected as a strong binding partner for CagA and is thought to support CagA spatial recruitment and presentation [94,95]. Binding interactions between substrate CagA and the T4CP Cagβ and between CagZ and Cagβ were also observed [91]. Deletion of *cagZ* prevents CagA translocation and decreases the cellular concentration of Cagβ. Complex formation of Cagβ with CagZ is likely to provide a stabilized receptor for effector protein recognition (figure 4*b*). Effective presentation of CagA is in turn facilitated by the chaperone CagF.

### (e) Substrate-receptor docking

In addition to substrate-binding chaperones, and factors that bind and stabilize the T4CP, another functional class of accessory proteins involved in substrate selection is particularly apparent in DNA transfer systems. Nucleoprotein substrate processing and presentation in these systems clearly differs in that—in addition to displaying a TS—the secreted protein needs to be associated with a specific genomic element for the process to be biologically meaningful. The auxiliary factors of conjugative relaxosomes bind to their cognate DNA sequences with exquisite specificity [96]. Their function extends to facilitating enzymatic processing of the T-strand in preparation for transfer (figure 4*a*). The higher order architecture of the relaxosome additionally has a key function in productive docking with the T4CP (figure 4*b*). In F-like systems, the plasmid-encoded relaxosome component TraM provides a crucial bridge between T4CP TraD and the plasmid. Highly specific binding to the cognate *oriT* controls selection of the plasmid in addition to the specific TS of the secreted relaxase. The recent crystal structure of a TraM–DNA complex revealed a unique cooperative binding mechanism that is key to TraM's selective bridging function [97]. The mechanism leaves much of the protein available to interact with TraD. Recruitment of the plasmid to the T4CP requires specific interaction between the TraM tetramerization domain and the C-terminus of TraD [86,97–99]. Flexibility in both proteins is probably important to coupling the plasmid to TraD over multiple contact points. The latter point is expected to be highly relevant to downstream mechanisms of transfer initiation. Consistent with that notion, *oriT* DNA is highly contorted in relaxosomes generally, and occupation of various binding sites by multiple architectural proteins brings the T4CP, the bridging protein and the relaxase with both its TS and DNA processing enzymatic activities into close proximity for mutual modulation of protein function. Relaxase activity is typically stimulated by the auxiliary factors and in some cases enhancement by the T4CP is also known [100,101]. This level of resting state activity can be supported by the docked relaxosome in the absence of transfer. Yet the complex is poised to control the initiation pathway once transfer is triggered by a contact-dependent mechanism.

### (f) Contact-dependent activation: extracellular signalling

Direct cell–cell contacts are essential for T4SS-mediated transfer, but little is known about these interactions. Host-cell attachment by *Agrobacterium* is independent of the Ti plasmid and instead relies on chromosomally encoded gene products involved in the synthesis and/or localization of periplasmic β1,2 glucan. The T-pilus is predominantly composed of VirB2 pilin. Two-hybrid screens for *Arabidopsis thaliana* proteins that interact with VirB2 revealed three related proteins (BTI_1_–BTI_3_) and a membrane-associated GTPase as candidates for the plant receptor in direct contact with the T-pilus [102]. VirB5 localizes to the pilus tip, implying a specific adhesive function in plant cell interaction. Multiple Cag proteins, including core component CagY, are associated with Cag-T4SS pili and contact the β1 integrin receptors of host cells [103]. Attempts to identify specific bacterial cell surface receptors for conjugative pili have been largely fruitless. Genetic screens have identified a few *Escherichia coli* mutants affected in OM protein composition (*ompA*) or lipopolysaccharide elaboration that only modestly reduce the efficiency of interbacterial DNA transfer. The apparent lack of dedicated receptors suggests that a strategy of promiscuous surface contacts by conjugative pili has advantages. Consistent with this proposal, conjugative pili support the formation of biofilm by bacterial host cells on diverse surfaces. This function for conjugative pili is one of the several independent mechanisms promoting biofilm development in many bacterial species [104,105].

Retraction of the F-pilus does not require an obvious exogenous signal such as cell contact, but once contact is established, the retraction cycle brings a mating pair into close proximity [106]. Details of the next steps, which ultimately progress to the initiation of transfer, remain undefined. Extensive areas of electron dense material called ‘conjugation junctions’ are observed between the OMs of apposed pairs in many conjugative systems [75,107,108]. Whether or not the pilus provides a passive conduit for nucleoprotein transfer, or whether the core complex of F—or variants of this structure—have active roles remains controversial. Genetic exchange has been detected in (surface immobilized) F pairs when pili are prevented from retracting [109,110]. In any case, transmission of ‘activating’ exogenous signals to the central checkpoint in donor cells as a prerequisite to transfer start could involve all of these structures.

### (g) Translating extracellular triggers into action: signal processing

Steps triggering transfer initiation remain unclear. Logically, we can project at least two: a receptor complex processes the incoming signal and relays it to a master regulator, which is then activated to permit substrate entry to the secretion channel. Evidence thus far supports the model that the T4CP is a master regulator in this process. In systems where components are produced constitutively, secretion substrates, chaperones and the T4CP with accessory selection factors may be in continuous contact. If so, this stably docked complex would provide an ideal receptor for signals transmitted from the cell surface. A recent study with the F-like plasmid R1 delineated specific protein domains in a relaxosome–T4CP subcomplex involved in perception of extracellular signals and their conversion to activation of transport [111]. For this study, the phage R17, which uses F-like pili as adsorption organelles, was used as a source of exogenous signalling. Penetration of the host cell by the ssRNA genome, which is covalently linked at the 3′-end to a phage protein, exploits the T4S machinery, including the T4CP. Despite expectations that phage uptake would be uncoupled from DNA processing in the host, the role of TraD in nucleoprotein import showed parallels to mechanisms of secretion. The data support a model of transfer activation (figure 4*c*) in which the T4CP perceives the intracellular signal of a docked relaxosome and responds with stimulation of T-DNA processing by the relaxosome. The complex of processed substrate and T4CP is in turn competent to receive extracellular signals conveyed from phage or cellular contact. The functional response enables the nucleoprotein substrate to enter the T4SS. Whole cell assays using phage thus have exciting potential to investigate transmission of signals in the form of conformational changes and energy flow from the cell exterior to the cytoplasm and back as a consequence of the architecture and operation of T4SS.

### (h) Energizing transfer

The central role that TraD, a T4CP, plays in both R1 transport and in R17 phage infection underscores the versatility and indispensability that ATPases exhibit in T4SS. T4SSs rely on multiple ATPases, including T4CPs that recognize relaxase–DNA conjugates or substrate proteins and transport these across the IM, and proteins that power assembly of the pore and pili through ATP hydrolysis. Although much remains to be learned, through recent biochemical studies, themes regarding these ATPases are becoming apparent. For example, while many of these ATPases are integral membrane proteins, some partition between the cytoplasm and the IM, and may be efficiently recruited to the IM only in the presence of the conjugative machinery [112,113]. These membrane ATPases oligomerize, often functioning as hexamers, yet other oligomeric forms have been observed *in vivo* and *in vitro* [112,114–116]. The different oligomeric forms could represent steps along an assembly pathway, or conversion between oligomeric forms may represent a form of regulation. These proteins can fill complex roles, potentially performing distinct activities at different stages in conjugation. For example, an F TraC mutant can form mating pairs despite being deficient in pilus formation, but shows a dramatic reduction in transfer efficiency [113]. The implication is that TraC plays roles in both pilus generation and elongation and in DNA transport.

In a subset of systems, we see an additional ATPase, namely the helicase activity in the relaxase found in F, R1 and R100 TraI, and R388 TrwC. Recent structural and sequence analyses have shown that the F/R1/R100 TraI helicase has an unusual organization, consisting of tandem RecD-like domains. The N-terminal domain contains an ssDNA-binding activity, the C-terminal domain contains the actual ATP binding and hydrolysis motifs, and both are required for function. Although relatively few conjugative systems express relaxase–helicase fusions, the ATPase activity of the helicase is essential in these systems.

## 5. Perspectives for future research

The recent advances in genome research, imaging technology and structural biology have propelled our understanding of these fascinating systems to new levels. As genome comparisons provide comprehensive views of the bacterial genetic repertoire, we can better understand the coevolution of multiple secretion machines resident in the same host. Information of this kind opens the possibility to examine how heterologous exchange alters the mechanisms and significance of these systems. The capacity to determine structures of higher order protein complexes opens the field to detailed mechanistic questions. A central focus is to learn how the pore assembles and how assembly is regulated. Yet understanding the functional interaction network of so many components during the processes of assembly and substrate secretion remains a tremendous challenge. Key questions have not been answered. Are protein and DNA substrates conveyed to the channel of the core complex directly from the donor cytoplasm, or are these first transferred across the cytoplasmic membrane to enter the channel secondarily from the periplasm? If the latter case proved to be true, the two-step mechanism observed for *B. pertussis* toxin would represent a norm rather than an exception in type IV secretion. We have yet to determine if substrate transfer is active or passive, or to comprehend the bottlenecks to secretion. Addressing the challenging question of how extracellular signalling regulates secretion initiation, and the molecular and structural nature of this extracellular signal, will certainly push the limits of our current technology further. To move forward, we clearly need to apply a new range of functional and whole cell assays that reconstitute discrete steps in the secretion pathway. The use of phage has great potential to explore transmission of signals in the form of conformational changes and energy flow from the cell exterior to the cytoplasm [117]. Importantly, the range of molecular reporters that are compatible with secretion and active in compartments of the cell envelope has been dramatically expanded [25,109,118]. Breakthroughs that resolve central mechanistic questions through the innovative use of these tools seem imminent. This information in turn will be valuable not only for the prospect of structure-aided drug design to prevent type IV secretion in relevant environments, but also to exploit these systems for controlled applications. These aims include targeted gene therapy [119], improved plant transformation [120] and targeted killing of bacterial cells [121], to name a few. Thus, the current potential for discovery in the field of bacterial type IV secretion is most promising.

## Glossary

T4SS: type IV secretion systems
T4CP: type IV coupling protein
IM: inner membrane
OM: outer membrane
Mpf: mating pore formation
*oriT*: origin of transfer
T-strand: DNA strand destined for transfer
Ti: tumour-inducing
ssDNA: single-stranded DNA
dot: defect in organelle trafficking
icm: intracellular multiplication
Cag: cytotoxin-associated gene
Ptl: pertussis toxin liberation system
dsDNA: double-stranded DNA
NTD: N-terminal domain
NBD: nucleotide-binding domain
TS: translocation signal
BID: Bep intracellular delivery
